# Monitoring viral load for the last mile: what will it cost?

**DOI:** 10.1002/jia2.25337

**Published:** 2019-09-12

**Authors:** Brooke E Nichols, Sarah J Girdwood, Thomas Crompton, Lynsey Stewart‐Isherwood, Leigh Berrie, Dorman Chimhamhiwa, Crispin Moyo, John Kuehnle, Wendy Stevens, Sydney Rosen, EQUIP Health

**Affiliations:** ^1^ Department of Global Health School of Public Health Boston University Boston MA USA; ^2^ Health Economics and Epidemiology Research Office Department of Internal Medicine School of Clinical Medicine Faculty of Health Sciences University of the Witwatersrand Johannesburg South Africa; ^3^ GIS Mapping Department Right to Care Johannesburg South Africa; ^4^ National Health Laboratory Service Johannesburg South Africa; ^5^ Department of Molecular Medicine and Haematology Faculty of Health Sciences University of the Witwatersrand Johannesburg South Africa; ^6^ EQUIP Zambia Lusaka Zambia; ^7^ United States Agency for International Development Lusaka Zambia

**Keywords:** viral load scale‐up, patient monitoring, geospatial modelling, cost modelling, health systems, viral load

## Abstract

**Introduction:**

Routine viral load testing is the WHO‐recommended method for monitoring HIV‐infected patients on ART, and many countries are rapidly scaling up testing capacity at centralized laboratories. Providing testing access to the most remote populations and facilities (the “last mile”) is especially challenging. Using a geospatial optimization model, we estimated the incremental costs of accessing the most remote 20% of patients in Zambia by expanding the transportation network required to bring blood samples from ART clinics to centralized laboratories and return results to clinics.

**Methods:**

The model first optimized a sample transportation network (STN) that can transport 80% of anticipated sample volumes to centralized viral load testing laboratories on a daily or weekly basis, in line with Zambia's 2020 targets. Data incorporated into the model included the location and infrastructure of all health facilities providing ART, location of laboratories, measured distances and drive times between the two, expected future viral load demand by health facility, and local cost estimates. We then continued to expand the modelled STN in 5% increments until 100% of all samples could be collected.

**Results and Discussion:**

The cost per viral load test when reaching 80% patient volumes using centralized viral load testing was a median of $18.99. With an expanded STN, the incremental cost per test rose to $20.29 for 80% to 85% and $20.52 for 85% to 90%. Above 90% coverage, the incremental cost per test increased substantially to $31.57 for 90% to 95% and $51.95 for 95% to 100%. The high numbers of kilometres driven per sample transported and large number of vehicles needed increase costs dramatically for reaching the clinics that serve the last 5% of patients.

**Conclusions:**

Providing sample transport services to the most remote clinics in low‐ and middle‐income countries is likely to be cost‐prohibitive. Other strategies are needed to reduce the cost and increase the feasibility of making viral load monitoring available to the last 10% of patients. The cost of alternative methods, such as optimal point‐of‐care viral load equipment placement and usage, dried blood/plasma spot specimen utilization, or use of drones in geographically remote facilities, should be evaluated.

## 1 Introduction

Routine annual HIV viral load monitoring is recommended by the World Health Organization for monitoring patients on antiretroviral treatment (ART) 1. While there has been progress in scaling up viral load programmes in many countries in sub‐Saharan Africa, most countries are not yet able to provide testing access to more than 50% of patients in need 2. For countries to expand access to HIV treatment services, and to move beyond current coverage targets towards full access, scaling up of the viral load monitoring system to reach even the most remote patients will be required.

Zambia, a lower middle‐income country in southern Africa with an estimated 1.2 million people living with HIV, is currently investing in scaling up its viral load monitoring programme 3. In 2018, approximately 40% of an estimated 800,000 patients on ART received a viral load test 4, 5. Current targets call for reaching 80% of patients by 2020 4, 6 and 90% coverage soon thereafter, to allow monitoring of UNAIDS 90‐90‐90 targets.

One barrier to broader access to viral load monitoring in Zambia is sample transportation. A total of 1475 facilities in Zambia provide ART and clinical care, ranging from 1 to 20,000 ART patients served. These ART patients require a minimum of one viral load test per year. Blood/plasma samples from patients at these facilities are collected by an *ad hoc* network of government, non‐governmental organizations, and private transportation contractors, and transported from facilities to one of 19 central referral laboratories in a network. Each lab in this network is equipped with polymerase chain reaction (PCR) – based equipment for assaying viral loads. The frequency of transportation of samples from clinics to referral laboratories ranges from daily to weekly to less than weekly, and is constrained by district and political boundaries and geographic challenges. The limited percentage of ART patients accessing viral load is, in part, due to sample transport challenges. A full description of the current system has been previously described 6. To address the lack of nationally coordinated sample transportation, we developed a geospatial model to design an optimized sample transportation network (STN) to provide daily or weekly access to achieve the Ministry of Health's goal of reaching 80% access by 2020 6. The final baseline optimized transport system model reaches 91% of ART patients nationally.

**Figure 1:**
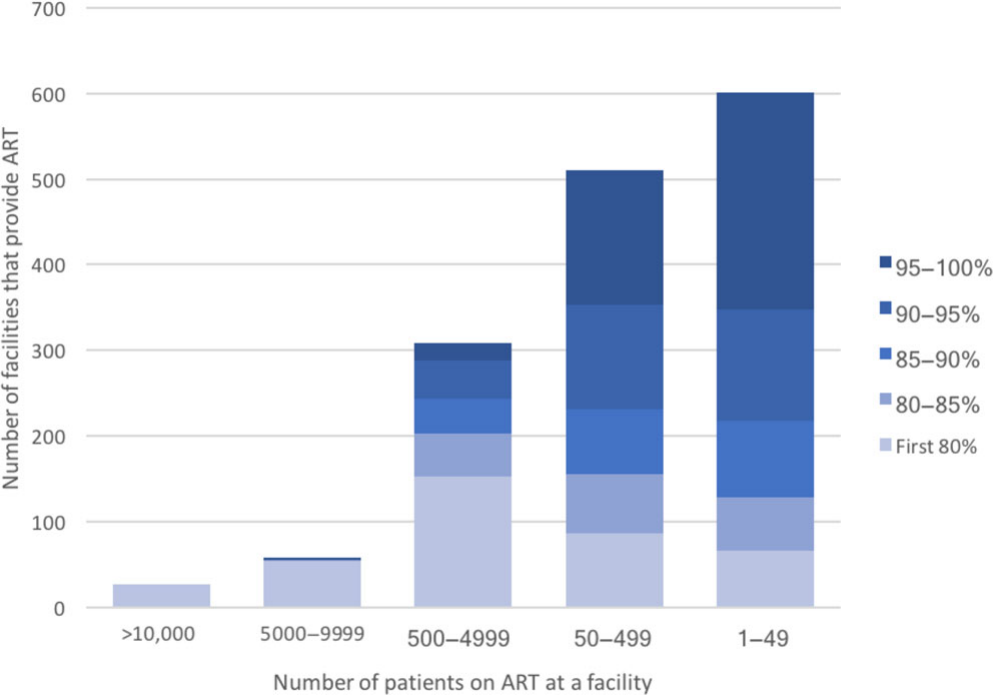
Distribution of all Zambian HIV‐treatment facilities by ART patient load and strata of access 5.

As illustrated in Figure 1, a large proportion of facilities provide care to modest numbers of ART patients. The smallest clinics (which are also the most numerous) are predominantly in the most rural locations and separated by the furthest distances to the nearest of the 19 referral laboratories (average drive‐time of facilities that provide care to <50 patients is 125 minutes to the nearest laboratory compared to 46 minutes at facilities that provide care to more than 5000 patients). Delivering any healthcare services to the last mile is typically expensive, as distances are usually long and patient volumes low. Economies of scale in expanding viral load access will increase to a point, but costs should be expected to increase again before full access is reached due to incremental infrastructure investments and increase travel distances to reach remote areas 7.

Using our geospatial optimization model, we estimated the incremental cost of scaling up viral load monitoring access to achieve 100% coverage of patients, both to inform policymakers and funders of the estimated cost of full viral load access using the current scale‐up strategy and to determine the point at which the cost‐per‐test begins to increase, suggesting the point at which alternative strategies should be considered. Importantly, we report the cost of expanding an optimized system. Increasing the reach of the system as currently implemented will be greater.

## 2 Methods

We developed and previously described a geospatial model that utilized a range of data to minimize transport distances and driving times, numbers of vehicles and costs required for viral load sample transport 6. The model, which is described in detail in our previous article, includes GPS coordinates of all HIV‐treatment facilities and laboratories, the expected viral load demand at each facility in 2020 5, 8, the actual recorded drive time and driving distance from each facility and hub to the testing laboratory, and the costs associated with sample transportation and laboratory viral load testing.

### 2.1 Coverage targets

We determined the cost of full coverage, such that all ART patients have access to viral load testing. For this purpose, we started by calculating the sample transport costs for the first 80% of patient volumes, which is the Ministry of Health 2020 coverage target. We then increased patient coverage by 5% increments up to 100% coverage.

### 2.2 Transport routing

Vehicle routing was determined using an ArcGIS Network Analyst tool 9, 10, 11, which optimized a set of transport routes to reach the set coverage targets, taking into account expected sample volumes, distance from the viral load laboratory, or transport hub to the facility, actual drive times and practical service delivery constraints (i.e. maximum kilometres driven in a day, working hours of a driver). The tool utilizes a heuristic process to minimize the objective function of reducing travel time and driving distance, within given constraints. The system was initially constrained to reach 80% of patients. We then allocated the STN incrementally more transport routes until 85%, 90%, 95% and 100% viral load coverage was achieved, using the same methodologic approach for each additional level of coverage.

### 2.3 Cost analysis

Annual recurrent costs for sample transportation were calculated for the original model and reported in 2018 USD 6. Costs include vehicle running costs incurred per kilometre of travel (fuel, maintenance and insurance), annualized vehicle and motorbike capital costs and personnel costs of operating the system including drivers and management personnel. We also estimated the full cost of a viral load test performed at a central laboratory to put the cost of sample transportation into perspective: we assumed that in expanding a centralized system, the cost per test will remain constant at the laboratory, while the cost of transporting the sample to the laboratory will vary. We then calculated the average cost per viral load sample transported for each level of coverage (total cost of the system divided by the total number of tests done in the system), as well as incremental cost per viral load sample for each level of coverage (the total cost of each increment, divided by the number of viral loads conducted in that increment).

### 2.4 Sensitivity analysis

A one‐way sensitivity analysis of individual cost inputs (reduction in the price of a viral load, exchange rate changes (± 20%), price of diesel increase and doubling the working life of a vehicle/motorbike) and of the assumed rate of ART scale‐up to 2020 (± 20%) was conducted to determine the impact of each of these parameters on our results.

## 3 Results and discussion

**Figure 2:**
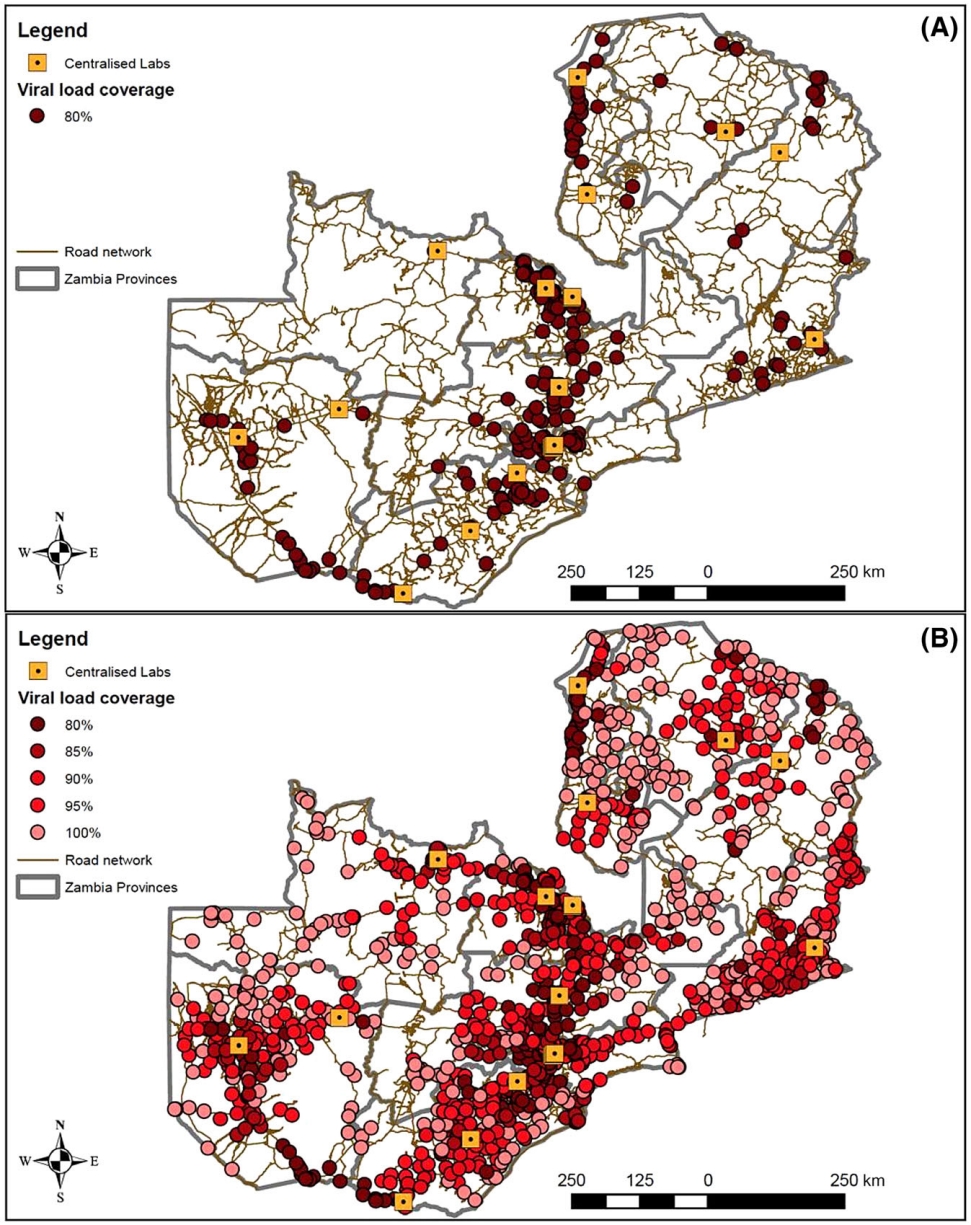
Viral load coverage in Zambia at 80% of patient volumes (A) and 100% of patient volumes (B).[Colour figure can be viewed at wileyonlinelibrary.com]

Our modelling results show that providing viral load access to the first 80% of patients can be reached by providing sample transport to just 355 (24%) health facilities (Figure 2A). To expand from 80% to 85% coverage, an increase of nine additional vehicles was required and a driving distance of 4036 km per week predicted. To expand from 85% to 90%, an increase of five additional vehicles was required and a driving distance of 7876 km per week predicted. To further expand from 90% to 95%, a much larger increase of 28 additional vehicles was required and a driving distance of 34,233 km per week was needed. Finally, to achieve true 100% coverage, an additional 33 vehicles, an additional 123 motorbikes, and an additional driving distance of 13,139 km per week were needed (Table 1, geospatial modelling results). Figure 2B illustrates the distribution of all ART treating facilities that correspond to 100% of the patient volume.

**Table 1:** Sample transportation network for the final 20% of viral loads: facilities reached, distanced travelled and vehicle requirements

Patients reached	Facilities reached	Total weekly distance travelled (km)	Number of vehicles required	Number of motorbikes required
Reaching 80%	355	50,119	45	12
Reaching 85%	538	54,155	54	12
Reaching 90%	744	62,031	59	12
Reaching 95%	1041	96,264	87	12
Reaching 100%	1475	109,403	120	135

As expected, the substantial increase in required resources after 90% scale‐up is also reflected in the cost per test. When reaching 80% of patients, the total cost per test is $18.99, with just 9% of the cost attributable to sample transport (Table 2). When reaching 85% of patients, the average cost per test increases slightly to $19.07, of which 10% of the cost is for sample transport. There is a large increase in incremental cost per viral load test in moving from 90% to 95% coverage, however, to $31.57 per sample, of which 45% is attributable to sample transport. The costs increase even more dramatically for the final five percent, with the incremental cost per test ballooning to $51.95, and sample transport comprising 67% of the cost of the viral load test. To prevent the increase in expected incremental cost per test, alternative strategies for reaching the final 10% should be explored.

**Table 2:** Cost of the sample transportation network for the final 20% of viral loads

Patients reached	Number of viral load tests expected: 2020	Total annual cost of sample transport	Cost per viral load at centralized laboratory	Average cost per test (percent of test due to sample transport)	Incremental cost per test (percent of test due to sample transport)
Reaching 80%	1350,189	$2386,571	$17.22	$18.99 (9%)	$19.70 (13%)
Reaching 85%	1440,197	$2663,313		$19.07 (10%)	$20.29 (15%)
Reaching 90%	1537,243	$2983,479		$19.16 (10%)	$20.52 (16%)
Reaching 95%	1641,863	$4484,773		$19.95 (14%)	$31.57 (45%)
Reaching 100%	1704,627	$6664,806		$21.13 (19%)	$51.95 (67%)

In 2020, the budget of Zambia's national ART programme is expected to be $253,000,000 12. An expansion of the viral load system to reach 100% of patients in 2020 would require a further $4278,806 (1.7%) increase in the national ART programme budget for sample transport and a $6103,425 (2.4%) increase for the viral load tests to be conducted at a centralized laboratory. The cost of the viral load tests themselves are greater than the additional cost of sample transport, but the fraction of viral load cost attributed to sample transport more than doubles from 9% to 19% from 80% to 100% access respectively.

Our results were most sensitive to a reduction in expected viral load volumes in 2020. If 20% fewer viral loads are run in 2020 than we report in our primary analysis, the incremental cost per sample transported for the 90% to 95% increment increases from $31.57 to $35.16, and for the 95% to 100% increment from $51.95 to $60.64. The total cost of sample transport remained the same, but the total cost was divided by a fewer number of viral load tests. Our results were next most sensitive to changes in the exchange rate. A strengthening of the Zambian kwacha to the US dollar by 20% resulted in higher overall costs of the sample transport network. This would result in an increase of the cost per sample transported of the 90% to 95% increment from $31.95 in our primary analysis to $33.97, and an increase of the 95% to 100% increment from $51.95 to $57.57.

The cost per viral load test estimated here follows a pattern that has been described for costs of other HIV‐related services. In most cases, there is a U‐shaped cost curve: the cost of starting up the programme is incrementally expensive for the initial patients, followed by large reductions in average cost for the largest percentage of patient volumes, and finally an incrementally expensive final portion of the programme 7, 13. Other studies examining the cost of scaling up HIV‐related programmes have described the economies of scale that come with implementation 14, 15, but they have not considered the cost to reach full 100% scale‐up. The high cost to reach the last mile is not surprising based on previous studies. Because current international targets call for only 80% to 90% coverage, however, not 100%, this final segment of the population is rarely considered. Consideration for this final segment of the population will become crucial for eventual HIV epidemic control.

Our model has several limitations. First, the number of viral load tests expected represent an absolute maximum number of tests to be conducted in 2020, based on expected ART scale‐up 8. While this is likely an overestimate, particularly in the short‐term, ideal maximum viral load volumes were taken so that the system would be able to handle these volumes and be optimized to future volume. While a uniform overestimation in the number of people on ART may alter the cost per test, it does not change the magnitude of the difference between scaling up from 80% to 100%. Second, the model is based entirely on technology that is currently used and approved for use in Zambia. As such, it does not include the use of dried blood/plasma spots or alternative strategies for specimen collection. If dried blood spot testing were to become available, they would be expected to alter the costs for the last 10% in particular, as transport would be required less frequently and samples could be stored at room temperature for weeks (about six weeks) at a time 16, There are, however, drawbacks to dried blood spots, including higher misclassification of results and higher limits of detection. Alternatively, small unmanned aerial vehicles (drones) capable of reaching rural areas and transporting small weight packages over the distance of up to 100km could be considered for transport of dried blood spot cards. Point‐of‐care devices, also not yet approved for local use, may also alter the cost per viral load test for the final 10% of patient volumes. Importantly, however, point‐of‐care tests tend to be cost‐effective only when testing volume is high and the point‐of‐care equipment has high utilization 17. Further investigation into these testing modalities and platforms is warranted. If reductions in costs and improved access across the viral load value chain using these alternative scenarios is demonstrated, Zambia's Ministry of Health and its partners may have an incentive to implement alternative strategies.

## 4 Conclusions

Given finite resources, methods to ensure low incremental costs for monitoring patients on ART are important. Continuing to scale up viral load to the last 10% of patients using the same approach that was used to scale‐up to the first 90% of patients will be costly. Investigation into novel approaches to reach the last 10% of patients in an affordable way is warranted to ensure equitable viral load access.

## Competing interests

The authors declare that no competing interests exist.

## Authors’ contributions

BEN conceived the study. BEN, SJG, TC, LS‐I, LB, JK, CM, DC and WS acquired and analysed data for the model. BEN, SJG and TC developed the model. BEN, SJG and SR interpreted model results. BEN and SR wrote the first draft of the manuscript. BEN, SJG, TC, LS‐I, LB, DC, CM, JK, WS and SR contributed to the writing of the manuscript. BEN, SJG, TC, LS‐I, LB, DC, CM, JK, WS and SR read and approved the final manuscript.
